# Structural host-microbiota interaction networks

**DOI:** 10.1371/journal.pcbi.1005579

**Published:** 2017-10-12

**Authors:** Emine Guven-Maiorov, Chung-Jung Tsai, Ruth Nussinov

**Affiliations:** 1 Cancer and Inflammation Program, Leidos Biomedical Research, Inc. Frederick National Laboratory for Cancer Research, National Cancer Institute, Frederick, MD, United States of America; 2 Sackler Inst. of Molecular Medicine, Department of Human Genetics and Molecular Medicine, Sackler School of Medicine, Tel Aviv University, Tel Aviv, Israel; NYU, UNITED STATES

## Abstract

Hundreds of different species colonize multicellular organisms making them “metaorganisms”. A growing body of data supports the role of microbiota in health and in disease. Grasping the principles of host-microbiota interactions (HMIs) at the molecular level is important since it may provide insights into the mechanisms of infections. The crosstalk between the host and the microbiota may help resolve puzzling questions such as how a microorganism can contribute to both health and disease. Integrated superorganism networks that consider host and microbiota as a whole–may uncover their code, clarifying perhaps the most fundamental question: how they modulate immune surveillance. Within this framework, structural HMI networks can uniquely identify potential microbial effectors that target distinct host nodes or interfere with endogenous host interactions, as well as how mutations on either host or microbial proteins affect the interaction. Furthermore, structural HMIs can help identify master host cell regulator nodes and modules whose tweaking by the microbes promote aberrant activity. Collectively, these data can delineate pathogenic mechanisms and thereby help maximize beneficial therapeutics. To date, challenges in experimental techniques limit large-scale characterization of HMIs. Here we highlight an area in its infancy which we believe will increasingly engage the computational community: predicting interactions across kingdoms, and mapping these on the host cellular networks to figure out how commensal and pathogenic microbiota modulate the host signaling and broadly cross-species consequences.

## Introduction

Rather than existing as independent organisms, multi-cellular hosts together with their inhabiting microbial cells have been viewed as “metaorganisms” (also termed superorganisms or holobionts) [1]. Millions of commensals, symbiotic, and pathogenic microorganisms colonize our body. Together, they comprise the “microbiota”. Microbiota are indispensable for the host, as they contribute to the functioning of essential physiological processes including immunity and metabolism. Hosts co-evolved with the microbiota. While some commensals are beneficial (symbionts), others may become harmful (pathobionts) [2, 3]. Microbiota help in immune system development. The immune system recognizes antigens of microorganisms e.g. DNA, RNA, cell wall components, and many others, through pattern recognition receptors, such as Toll-like receptors (TLRs) and downstream intracellular signaling circuitries are activated to generate immune responses [4]. However, like self-antigens, antigens from commensal microbiota are tolerated with no consequent inflammatory responses. This makes gut microbiota accepted as “extended-self” [5]. Still, under some circumstances, commensals may act as pathogens. For example, *Staphylococcus aureus* [6] or *Candida albicans* [7] are commensals of human, but in “susceptible” hosts, they can undergo commensal-to-pathogen transition. Thus, identifying microorganisms that reside in the host, and within these, those that are responsible for distinct host phenotypes, and the host pathways through which they act are significant goals in host-microbiota research. Microbiota survival strategies within the host are likely to be limited. Analysis of their repertoire may reveal core modules, thereby helping in classification, mechanistic elucidation and profile prediction. Here we provide an overview of structural host-microbiota interaction networks from this standpoint.

### The host-microbiota interactions in the metaorganism

The host interacts with microbiota through proteins, metabolites, small molecules and nucleic acids [8, 9]. The microbiota employs a range of effectors to modulate host cellular functions and immune responses. They have sophisticated relationships with the host, and network representation enables an effective visualization of these relationships [10]. Most proteins of bacterial and eukaryotic pathogens are not accessible to bind to host proteins; but some of their proteins either bind to host surface receptors [11] or enter the host cell and interact with host cytoplasmic proteins. Various bacterial species have a secretion system–a syringe-like apparatus–through which they inject the bacterial effectors directly into the host cell cytoplasm [12]. Via HMIs, they specifically hone in on key pathways, alter host physiological signaling, evade the host immune system, modify the cytoskeletal organization [13, 14], alter membrane and vesicular trafficking [2, 11, 13], promote pathogen entry into the host, shift the cell cycle [15, 16], and modulate apoptosis [17]. All are aimed to ensure their survival and replication within the host. Host signaling pathways that are targeted by microbiota and turned on or off may change the cell fate. Unraveling the HMIs for both commensals and pathogens can elucidate how they repurpose the host signaling pathways and help develop new therapeutic approaches.

**Fig 1:**
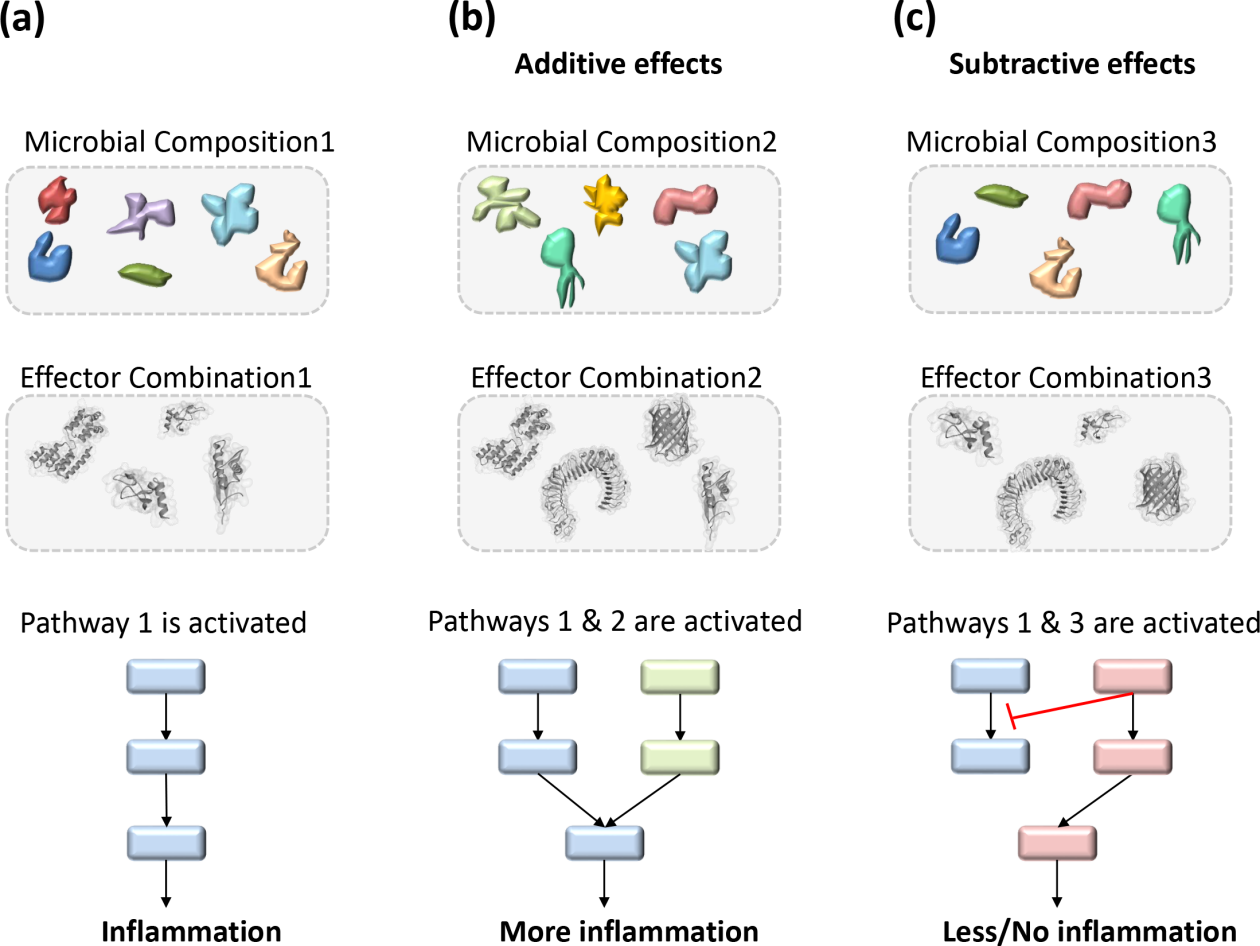
Distinct microbial compositions may lead to different outcomes (hypothetical scenario). Combinatorial effects of microbial effectors and the active host pathways determine the cell response. **(a)** Composition1 has certain microorganisms that secrete effector protein combination1. These effectors activate pathway1 in the host, which produces pro-inflammatory cytokines. **(b)** Composition2 secretes effector combination2 and activates pathway2 in addition to pathway1. Additive effects of these two pathways amplifies the signal and promotes inflammation (cross-activation). **(c)** Microbial composition3 utilize effector combination3 to activate both pathway 1 and 3, which have opposing outcomes. Subtractive effects of these pathways result in no inflammation (cross-inhibition).

HMIs have complex and dynamic profiles. Studies often focus on individual protein interactions and try to explain the pathogenicity of a microorganism with a single interaction. However, considering host-microbiota interactions one-at-a-time may not reflect the virulence scheme [18]. For instance, replication of vaccinia virus necessitates the establishment of a complex protein interaction network [19] and hence focusing on only one HMI is incomplete and may be misleading. At any given time, hundreds of different species reside in the gut. Different microbial compositions and hence effector protein combinations from these microbial species may have additive (cross-activation) or subtractive (cross-inhibition) [4] impacts on the host pathways, which lead to signal amplification or inhibition, respectively (Fig 1).

Since numerous bacteria will be sensed by the host immune system at any given time, more than one signaling cascade will be active in a cell. Communication and crosstalk among active, or active and inhibited, pathways determine the ultimate cellular outcome [4]: to survive, die, or elicit immune responses. The combinatorial ramifications of all active (or suppressed) host pathways and HMIs will be integrated to shape the type and magnitude of the response, and thus the cell state. To tackle the pathogenicity challenge, it is reasonable to concomitantly consider all host pathways and HMIs. The transkingdom (metaorganism) network analysis is a robust research framework that considers host and microbiota as a whole [1]. Systems biology approaches that integrate the HMIs with host endogenous protein interaction networks reveal the systematic trends in virulence strategies of pathogens.

### Host-microbiota network core modules

Here we ask how interspecies (superorganism) networks can facilitate the understanding of the role of microbiota in disease and health. We focus on host-microbiota protein interaction networks since many bacteria or virus-induced pathological processes require physical interactions of host and microbial proteins [20]. The availability of genome-wide high throughput omics data makes it possible to associate microbiota with certain host phenotypes at multiple levels and construct host-pathogen interaction networks at the transcriptome [21], proteome [22], and metabolome levels [23]. Steps toward the construction of host-microbiota networks of gene [1], mRNA [24], protein-protein interaction (PPI) [25–28], and metabolic networks [29] have already been taken. Within this framework we highlight molecular mimicry, a common strategy that microorganisms exploit to bind to host proteins and perturb its physiological signaling. Mimicry of interactions of critical regulatory nodes in core network modules in the immune system, may be a major way through which pathogens adversely subvert–and commensal microbiota may beneficially modulate–the host cell.

### Molecular mimicry

Microbiota developed several strategies to interact with host proteins and modulate its pathways. One efficient way is molecular mimicry, which has been extensively reviewed in our recent study [9]. Molecular mimicry can take place at four levels: mimicking (i) both sequence and 3D structure of a protein, (ii) only structure without sequence similarity, (iii) sequence of a short motif–motif mimicry, and (iv) structure of a binding surface without sequence similarity–interface mimicry. Interface mimicry (protein binding surface similarity) seems to be the most common type of molecular mimicry. Global structural similarity is much rarer than interface similarity both within and across species. Thus, employing interface mimicry instead of full-length sequence or structural homology allows microbes to target more host proteins. Molecular mimicry follows the principle suggested over two decades ago that proteins with different global structures can interact in similar ways [30–32].

**Fig 2:**
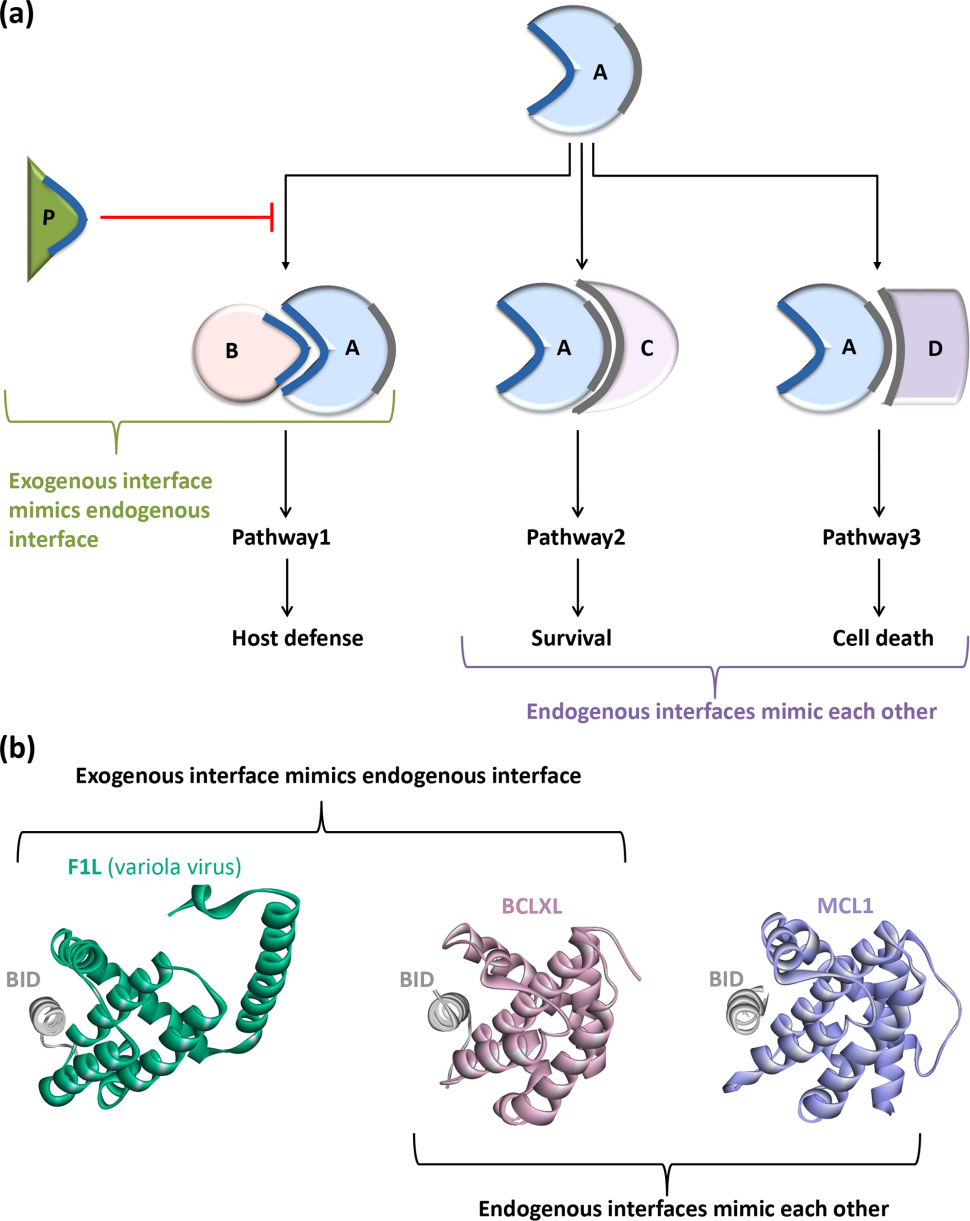
Endogenous (intra-species) and exogenous (inter-species) interface mimicry. **(a)** A, B, C, D are host proteins and P is pathogenic protein. Protein A has two interfaces: through blue interface it binds to B and through grey interface it binds to C and D. C and D proteins employ similar interfaces to bind to A. So, endogenous interfaces mimic each other. Pathogenic protein P has similar interface as B and competes to bind to the blue interface on A. In this case, an exogenous interface mimics an endogenous interface. **(b)** The F1L protein of variola virus interacts with human BID protein (5ajj:AB.pdb) and inhibits apoptosis in the host cell by hijacking the interface between human BID-BCLXL (4qve:AB.pdb): an exogenous interface mimicking an endogenous one. Human MCL1 protein binds to human BID (5c3f:AB.pdb) in a very similar fashion that BCLXL does: endogenous interfaces mimicking each other.

Interface mimicry is frequently observed within intra- [33–35] and inter-species [18, 36] (Fig 2) (intra-species interface mimicry: distinct proteins from the same species having the same/similar interfaces; inter-species interface mimicry: proteins from different species hijack the same interface architectures). Interface similarity allows proteins to compete to bind to a shared target. If an interface is formed between proteins from the same species, it is an ‘endogenous interface’. If it is formed by proteins from two different species, it is an ‘exogenous interface’ [18, 36]. Endogenous (intra-species) interfaces mimic each other [33–35], and exogenous (inter-species) interfaces mimic endogenous interfaces (Fig 2) [18, 36]. By mimicking endogenous interfaces, exogenous interfaces enable pathogenic proteins to compete with their host counterparts and hence rewire host signaling pathways for their own advantage [9]. They can either inhibit or activate a host pathway. For example, the *Helicobacter pylori* secreted protein CagA interacts with human tumor suppressor TP53BP2, inhibits apoptosis and allows survival of infected host cells [37]. However, Map protein of *E*. *coli* and SopE protein of *Salmonella* bacteria bind and activate human Cdc42, a Rho GTPase, and trigger actin reorganization in the host cell, facilitating bacterial entry into the host [38].

### An example of microbial subversion of a major pattern recognition receptor family: TLR family

One of the most significant pattern recognition receptor families in the innate immune system is the TLR family. Its members detect diverse bacterial compounds, like peptidoglycan, lipopolysaccharide, and nucleic acids of bacteria and viruses. They induce pro-inflammatory or anti-viral responses. Once activated, they recruit other TIR-containing proteins such as Mal and MyD88 or TRAM and TRIF through their cytoplasmic TIR domains, forming the MyD88- and TRIF-dependent TIR domain signalosomes, respectively [39]. MyD88 also assembles into a Myddosome structure through its death domain together with IRAK4 and IRAK1/2 death domains. The myddosome then recruits E3 ubiquitin ligases–either TRAF6 or TRAF3 –to catalyze the addition of K63-linked ubiquitin chains to themselves, which serve as a docking platform for other proteins to bind, such as TAK1. Subsequently, NF-κB and MAPK pathways are activated. In the NF-κB pathway, TAK1 phosphorylates and activates IKK. Activated IKK in turn phosphorylates IκB, which is the inhibitor of NF-κB. Phosphorylated IκB is then ubiquitylated by other E3 ubiquitin ligases (K48-linked ubiquitin chain) and targeted for proteosomal degradation. This liberates the p65 subunit of NF-κB to translocate to nucleus and initiate transcription. In the MAPK pathway, TAK1 serves as a MAP3K that activates ERK1/2, p38 and JNK pathways. The TRIF-dependent downstream path of TLRs recruits TRAF3 and leads to activation of interferon regulatory factors (IRFs) and production of key antiviral cytokines, interferons (IFNs).

**Fig 3:**
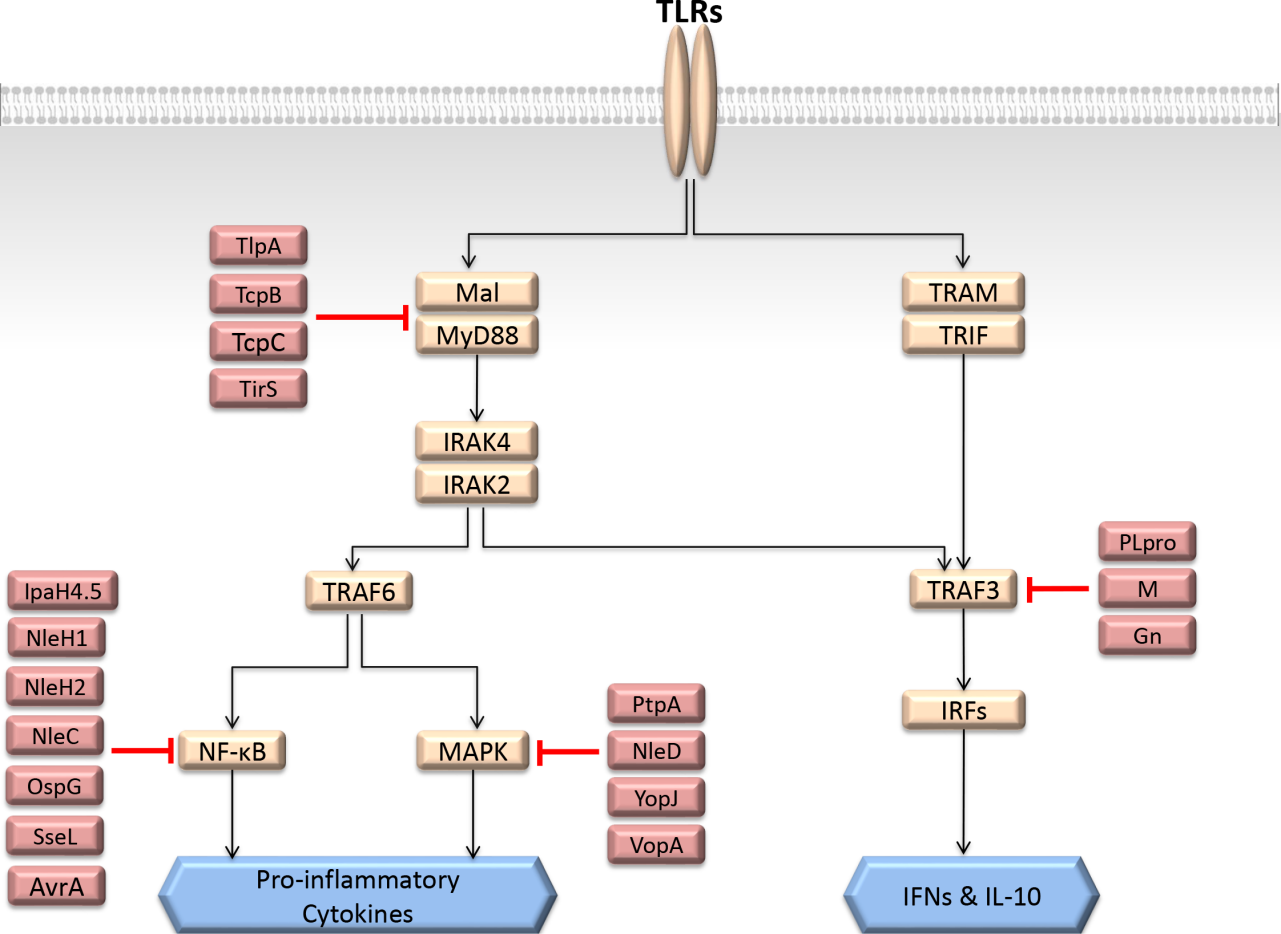
Subversion of TLR pathway by various bacterial and viral proteins at several steps. This is a simplified representation of TLR pathway where the orange nodes are the host proteins and red nodes are the microbial proteins.

The TLR pathway is regulated by several endogenous negative regulators to prevent excess inflammation [40]. Since this is one of the major immune pathways, its signaling is targeted by diverse microorganisms at various steps (Fig 3), which is broadly summarized in [41]. For instance, bacterial TIR-containing proteins such as TlpA of *Salmonella* [42], TirS of *Staphylococcus aureus* [43], TcpC of *E*. *coli* [44], and TcpB of *Brucella melitensis* [45], compete with endogenous TIR-containing proteins and interfere with the assembly of the TIR-domain signalosome and prevent downstream signaling. Since these microbial proteins do not enzymatically modify the endogenous proteins, elucidation of their inhibition mechanism requires structural information. The availability of the structures of their complexes with the orchestrators of the TLR pathway can clarify how they inhibit downstream signaling. Microbial proteases prevent both TLR-induced MAPK and NF-κB signaling and lead to proteosomal degradation of the key orchestrators in these pathways: NleD of *E*. *coli* cleaves JNK and p38, inhibiting MAPK pathway; and NleC cleaves p65, inhibiting NF-κB [46]. There are also bacterial acetyltransferases (VopA of *Vibrio parahaemolyticus* [47], YopJ/YopP of *Yersinia* [48], AvrA of *Salmonella Typhimurium* [49]), kinases (OspG of *Shigella* [50], NleH1 and NleH2 of *E*. *coli* [51]), phosphatases (PtpA of *Mycobacterium tuberculosis* [52]), ubiquitin ligases (IpaH4.5 of *Shigella* [53]), deubiquitylases (SseL of *Salmonella Typhimurium* [54]), and many more that inhibit either MAPK, NF-κB, or both pathways.

Since the TRAF3-dependent path gives rise to anti-viral immune responses, several viral proteins such as M [55] and PLpro [56] proteins of *SARS coronavirus*, and Gn of *NY-1 Hantavirus* [57, 58] inhibit this protein to limit IFN production [59].

Here, we listed only a couple of microbial proteins targeting TLR pathway as examples. There are many others. The TLR pathway does not constitute the whole innate immune system; other immune pathways also need to be considered as well as how these microbial proteins affect them as a whole. This can help foreseeing what kind of responses the coordinated actions of these pathways together with TLRs would generate.

### PPI networks and their topological properties

Most cellular processes are elicited by proteins and their interactions. Graph representations of PPI networks, where proteins are the nodes and their interactions are edges, are helpful for delineating the global behavior of the network. Topological features of networks, such as degree (number of edges), betweenness-centrality (how a node affects the communication between two nodes), lethality-centrality, hubs (proteins with high node-degree, i.e. several interaction partners), non-hubs (with only a few partners), and bottlenecks (nodes with high betweenness-centrality) help characterization of the importance of the nodes, i.e. the contribution of the node to network integrity [60, 61]. Early on, hubs were classified as either party or date hubs. While party hubs interact with many partners at the same time since they use distinct interfaces, date hubs interact with their partners one at a time due to their overlapping interfaces. To infer whether a hub is party or date hub, structural information (interface residues) [62] or gene expression data (co-expressed proteins have higher chances of interacting with each other) [63] were used. Later on, this definition was questioned. Among the reasons were the many examples where a protein node can serve concomitantly as a party and date hub. Large assemblies typically fall into this category.

Biological networks are often scale-free, with many non-hubs and fewer hubs [64, 65]. Not all nodes have the same effect on the network: random node attacks do not harm the network as much as removing hubs from scale-free networks [66]. Degree and betweenness-centrality are measures of the contribution of nodes to network integrity. There are also “essential” nodes, knock-out of which leads to lethality: a feature also known as “lethality-centrality”. Attack of a hub by microbiota is likely to influence the cell, either resulting in lethality, or in beneficial modulation. Thus, integrated superorganism interaction networks may suggest candidate host and microbial node targets. Structural interspecies networks and their topological features can shed light on how microbiota alter the host signaling and what will the outcome in different settings be.

### Structural metaorganism networks

**Fig 4:**
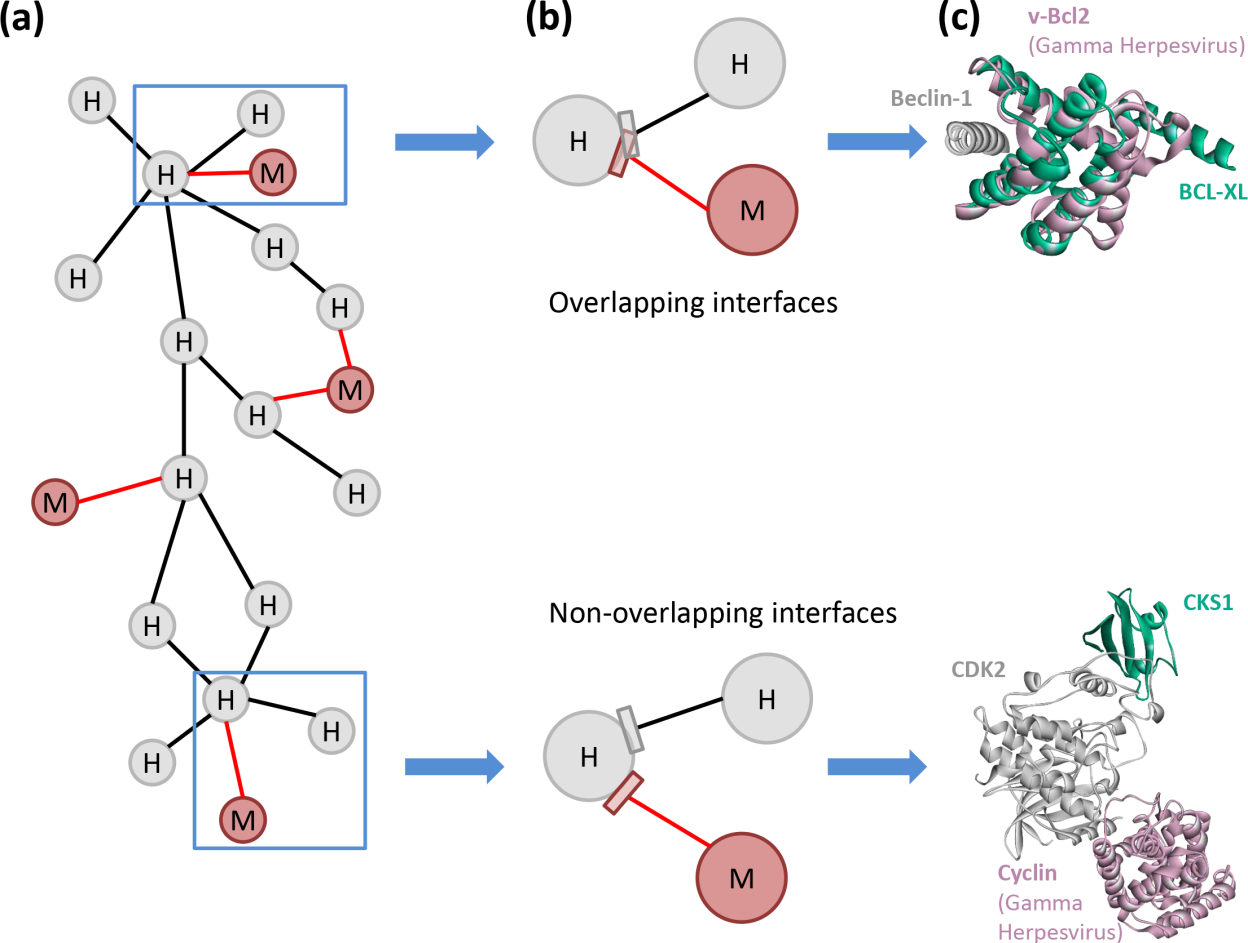
Structural HMI network. **(a)** High-resolution metaorganism network where grey nodes represent host proteins, red nodes microbial proteins, black edges host PPIs, and red edges HMIs. If an exogenous interface–HMI–(red edges) overlaps endogenous ones, it can abolish the endogenous PPI. If the exogenous and endogenous interfaces do not overlap, then the HMI does not disrupt the endogenous PPI. Without 3D structure knowledge of PPIs and HMIs, we cannot infer whether the interfaces overlap or not. **(b)** Zoom-in views of blue boxes show whether the interfaces overlap. **(c)** 3D representations of the interactions shown in part (b). Above diagram shows the superimposed view of 4mi8:AC and 2p1l:AB where Gamma Herpesvirus vBcl2 (red) and human Bcl-XL (green) binds to the same site on human Beclin-1 (grey). Here, an exogenous interface mimics an endogenous interface. Below diagram shows the superimposed view of 1f5q:AB and 1buh:AB, where Gama Herpesvirus Cyclin (red) and human CKS1 (green) bind to distinct interfaces on human CDK2 (grey).

Available HMI networks demonstrate that different bacteria often hijack the same host pathway in distinct ways [12], like the TLR pathway subversion by numerous microbial species (Fig 3). However, importantly, the same host pathway is often targeted at several nodes, which was suggested to guarantee modulation of cellular function [12]. Although there are a number of examples of constructed networks of host-pathogen superorganism interactions [12, 19, 67–75], there are many fewer attempts of integrating 3D structural data with the HMI networks [18]. Traditional network representation has low resolution, missing important details. However, structural interaction networks provide a higher resolution with mechanistic insights. They can decipher and resolve those that are not obvious in binary interaction networks [36]. The potential of structural networks in unraveling signaling pathways was demonstrated earlier [39, 40, 76, 77]. They are essential to fully grasp the mechanisms exerted by pathogens to divert the host cell signaling and attenuate immune responses. Fig 4 displays an example of a structural HMI network, showing how host PPIs can be affected by HMIs. Structures can detail which endogenous host PPIs are disrupted by the HMIs, possible consequences of mutations on either host proteins or pathogenic proteins, and whether variants of a virulence factor in different strains of the same species have distinct HMIs. For instance, the pro-35 residue on HIV accessory protein Vpr is at the interface with human CypA and its mutation to Alanine abrogates the interaction [78]. The structure of the CypA-Vpr complex shows that pro-35 is at the interface. If the structure of the Vpr-CypA complex was unknown, it would have been difficult to understand why, or how, this mutation disrupts the PPI.

Previously built structural HMI networks demonstrated that endogenous interfaces that are hijacked by pathogens are involved in multiple transient interactions [18, 36]. These endogenous interfaces exhibit ‘date-like’ features, i.e. they are involved in interactions with several endogenous proteins at different times [18, 36]. Hub and bottleneck proteins at the crossroads of several host pathways were suggested to be the major targets of viral and bacterial proteins [26, 28] and interface mimics allow transient interactions with the hub [79]. This allows them to interfere with multiple endogenous PPIs. It was proposed that microorganisms causing acute infections, which are dramatic for the host, are likely to interfere with the hubs, whereas others that lead to persistent infections tend to target non-hubs [80]. During acute infection, pathogens replicate very quickly and are transmitted to new hosts. However, during chronic infections, they adapt to the host environment, which allows them to reside there for a long period of time. Thus, how microbiota target certain proteins and pathways at the molecular level is of paramount importance.

Detecting the HMIs, mapping them onto networks and determining their 3D structures as a complex are the major steps to construct structural HMI networks. Despite the progress in experimental techniques, it is still challenging to determine structures of PPI complexes, particularly HMIs. Since large-scale experimental characterization of host-pathogen PPIs is difficult, time consuming, and costly, experimentally verified HMI data is scarce. It is important to note that available endogenous protein structures are biased towards permanent, rather than transient interactions. If majority of the HMIs are transient, this presents another hurdle since they will be under-represented in the structural space. Several HMI databases have been developed, such as PHISTO [81], HPIDB [82], Proteopathogen [83], PATRIC [84], PHI-base [85], PHIDIAS [86], HoPaCI-DB [87], VirHostNet [88], ViRBase [89], VirusMentha [90], HCVpro [91], and likely some others as well. However, these databases cover only a limited number of pathogens and their interactions. Given that thousands of species residing in the host, thousands of HMIs are yet to be identified. Computational approaches are becoming increasingly important in prioritizing putative HMIs and complementing experiments. Hence, construction of comprehensive metaorganism networks and increasing the coverage of the host-microbiota interactome will still mostly rely on computational models in the near future [92].

Computational modeling of intra-species interactions is a well-established area; detection of inter-species interactions is relatively new. Available computational tools to predict host-pathogen interactions have been recently reviewed by Nourani *et al*. [93]. Current methods mostly depend on global sequence and structure homology. Sequence-based methods focus only on orthologs of host proteins. However, sequence by itself is insufficient to detect the targets of pathogenic proteins because several virulence factors do not have any sequence homologs in human. For instance, the VacA protein of *Helicobacter pylori*, the most dominant species in gastric microbiota, has a unique sequence that does not resemble any human protein [94]. Still, it alters several host pathways [95]. With sequence-based methods, it is impossible to find HMIs for VacA. As noted above, global structural mimicry is much rarer than interface mimicry. Hence, utilizing interface similarity, rather than global structural similarity in a computational approach would generate a more enriched set of HMI data together with atomic details [9].

Several studies suggested that the available interface structures are diverse enough to cover most human PPIs [96–99]. Therefore, success of template-based methods for prediction of human PPIs is very high [34]. Since exogenous interfaces mimic endogenous ones, both available endogenous and exogenous interface structures can be used as templates to detect novel HMIs. Thanks to the rapid increase in the number of resolved 3D structures of human-pathogen PPIs in recent years [100] and advances in structural and computational biology, the performance of interface-based methods is expected to increase.

Both experimental and computational approaches have false-positives and false-negatives with varying rates depending on the approach. Although the coverage of interface-based methods is higher, their false-positive rate is also higher. Despite this, attempts to complete the host-microbiota interactome will improve our knowledge of microbiota and their roles in health and disease.

## Conclusions

Advances in host-microbiota research will revolutionize the understanding of the connection between health and a broad range of diseases. Building the rewired host-microbiota multi-organism interaction network, along with its structural details, is vital for figuring out the molecular mechanisms underlying host immune modulation by microbiota. Topological features of such networks can reveal the selection of host targets by the microbiota. Structural details are essential to fully grasp the mechanisms exerted by microbiota to subvert the host immunity. Identification of the HMIs will also help drug discovery and integrated superorganism networks would suggest how inhibition of an HMI can influence the whole system.

Here we highlighted the importance of building structural HMI networks. However, not only HMIs are important; although to date data are scant, crosstalk among microorganisms is also emerging as critical. Alterations in their population dynamics may lead to dysbiosis. Signals from gut microbiota resulting from population shifts can affect profoundly several tissues, including the central nervous system. Dysbiosis of microbiota is involved in several diseases, such as inflammatory bowel disease [101], autoimmune diseases (e.g. multiple sclerosis) [102], neurodegenerative diseases (e.g. Parkinson’s) [103], and cancer [104, 105]. Identifying bacterial effectors, or effector combinations, which are responsible for specific phenotypes, is challenging. In line with this, recently, Parkinson’s disease (PD) patients are found to have altered gut microbiota composition [106, 107]. Transplanted microbiota from PD patients, but not from healthy controls, induce motor dysfunction and trigger PD in mice. It is not clear however whether dysbiosis triggers PD or it arises as a consequence of the disease [103].

The role of microbiota in host health and disease might be even more complex than thought: Commensals once being benign can convert to disease-causing pathogens; different compositions of microbial communities trigger different phenotypes; more than one host pathway is targeted by more than one effector; the same microbial effector/antigen is sensed by several pattern recognition receptors (back-up mechanism, compensatory microbial sensing [4]) and genetic variation in hosts results in different responses (i.e. some commensals transition to pathogen only in “susceptible” individuals). Current knowledge on microbiota and their interactions with the host is still in its infancy, but given the advances that are accomplished so far and the attention this field started to attract these days, it is likely that many unknowns and questions will be uncovered soon.
